# Calibration and Algorithm Development for Estimation of Nitrogen in Wheat Crop Using Tractor Mounted N-Sensor

**DOI:** 10.1155/2015/163968

**Published:** 2015-02-24

**Authors:** Manjeet Singh, Rajneesh Kumar, Ankit Sharma, Bhupinder Singh, S. K. Thind

**Affiliations:** ^1^Department of Farm Machinery and Power Engineering, Punjab Agricultural University, Ludhiana 141001, India; ^2^Krishi Vigyan Kendra, Samrala, Punjab 141114, India; ^3^Krishi Vigyan Kendra, Mansa, Punjab 151505, India; ^4^Department of Botany, Punjab Agricultural University, Ludhiana 141001, India

## Abstract

The experiment was planned to investigate the tractor mounted N-sensor (Make Yara International) to predict nitrogen (N) for wheat crop under different nitrogen levels. It was observed that, for tractor mounted N-sensor, spectrometers can scan about 32% of total area of crop under consideration. An algorithm was developed using a linear relationship between sensor sufficiency index (SI_sensor_) and SI_SPAD_ to calculate the N_app_ as a function of SI_SPAD_. There was a strong correlation among sensor attributes (sensor value, sensor biomass, and sensor NDVI) and different N-levels. It was concluded that tillering stage is most prominent stage to predict crop yield as compared to the other stages by using sensor attributes. The algorithms developed for tillering and booting stages are useful for the prediction of N-application rates for wheat crop. N-application rates predicted by algorithm developed and sensor value were almost the same for plots with different levels of N applied.

## 1. Introduction

Precision agricultural practices have significantly contributed to the improvement of crop productivity and profitability. It enhances farm input use efficiency and reduces environmental impacts [1]. Today, precision agricultural practices are providing farmers with valuable information, enabling them to make the right decisions with respect to management of crop inputs such as fertilizer, seed, pesticides, and water. Among all Precision Crop Management activities, nitrogen management, which determines the optimal amount of nitrogen (N) for a specific location based on the yield potential, is the most frequently practiced operation. Efficient nitrogen fertilizer management can be defined as managing N fertilizer, so the crop uses as much of the applied nitrogen as possible each year [2].

Plants normally contain 1–5% nitrogen by weight. Nitrogen generally has more influence on crop growth, yield, and quality than any other nutrient commonly provided as fertilizer to crops. Many farmers often use uniform rates of N fertilizers based on expected yields (yield goal) that could be inconsistent from field-to-field and year-to-year depending on factors that are difficult to predict prior to fertilizer application. Also, farmers often apply fertilizer N in doses much higher than the blanket recommendations to ensure higher crop yields. Large temporal and field-to-field variability of soil N supply restricts efficient use of N fertilizer when broad based blanket recommendations are used [3, 4]. A mismatch between N supply and crop requirement can potentially hamper crop growth or harm the environment, resulting in low N use efficiency and economic losses. Plant N can be estimated from tissue sampling, chlorophyll meter measurements [5–7], and remote sensing [8–11]. Tissue sampling for nitrogen availability is well documented and requires considerable effort for sample collection and processing. In addition, results are not immediately available. Nitrogen fertility management encompasses four major components: source, placement, timing, and rate [12]. To accomplish this, producers must be aware of the various sources of N available to the crop other than fertilizer and how to minimize N loss. The total amount of N required must be determined from reasonable estimates of yield, residual soil nitrate-nitrogen, and soil organic matter followed by an evaluation of N credit from other sources such as irrigation water, legumes, and manure. Making accurate N fertilizer recommendations can improve fertilizer efficiency, reducing unnecessary input cost to producers and environmental impact of N losses. But it is very difficult for a farmer to have account of all these N-sources and losses. Measurement of real time N-uptake in plants may be a solution.

Recently, optical sensing of crop canopy spectral reflectance from ground, aircraft, and satellite-based platforms on Normalized Difference Vegetation Index (NDVI) has been proposed to identifying the crop nitrogen (N) deficient portions in the fields. These instruments has the potential to provide a fast, inexpensive, and accurate estimate of plant biomass production and grain yield prior to harvest, which would be beneficial for crop breeders [13, 14]. Martin et al. [15] found that NDVI increased with maize growth stage during the crop life cycle and a linear relationship with grain yield was best at the V7–V9 maize growth stages. This study also found that NDVI increased until the V10 growth stage when a plateau was reached and NDVI began to decrease after the VT growth stage. Shaver et al. [16] found that NDVI is highly related to leaf nitrogen (N) content in maize (*Zea mays* L.). Remotely sensed NDVI can provide valuable information regarding in-field N variability and significant relationships between sensor NDVI and maize grain yield have been reported.

Leaf color charts for proper N-management have been recommended in many countries but there are certain issues in their adoption by the farmers. Green seeker, Crop circle, Crop spec, and N-sensor are on-the-go sensing devices to achieve variable-rate application of N fertilizer according to site-specific field conditions. In the present study, the responses of N-sensor (Make Yara International) under variable levels of N availability were investigated for wheat crop.

The tractor mounted N-sensor is one of the sensor technologies which measures the level of light reflectance by crop canopies using spectrometers. But, there is a need to calibrate and develop an algorithm for N-application for locally recommended varieties of wheat. It can measure crop reflectance characteristics and calculate NDVI for development of algorithms to calculate optimal N application rates. In this paper, an algorithm is developed for converting sensor measurement into N-application. The sensor algorithm is based on a SPAD algorithm published by Varvel et al. [17] that outlines the procedure for translating SPAD reading into N-rate recommendation. The objectives of the present paper are

1. to calibrate the tractor mounted N-sensor for estimation of nitrogen and chlorophyll available in wheat crop;
2. to develop an algorithm for N-sensor based on SPAD meter algorithm.

## 2. Material and Methods

### 2.1. Experimental Planning

**Figure 1:**
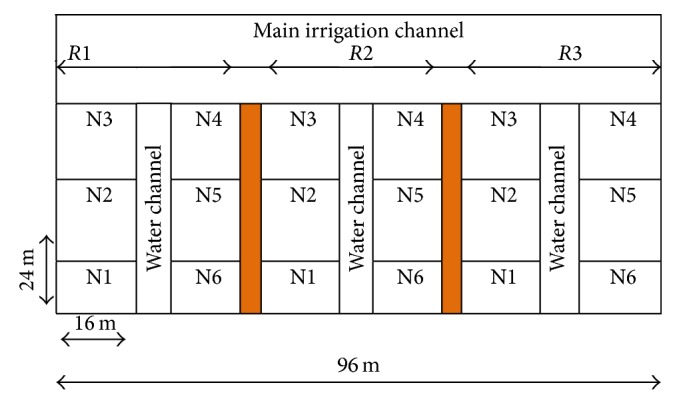
Field layout of the experiment.

Field experiments to investigate the tractor mounted N-sensor for wheat crop under variable nitrogen application rates were carried out at the Research Farm of the Department of Farm Machinery and Power Engineering, Punjab Agricultural University, Ludhiana. Soil of experimental field was sandy loam in texture and normal in pH, EC. For experiment, the university recommended PBW550 wheat variety was selected. It was raised with 6 different nitrogen levels, that is, 0, 30, 60, 90, 120, and 150 kg N/ha. The crop was sown on 21 November 2012. The experiment was carried out in randomized complete block design with three replications (Figure 1). The nitrogen in form of urea (46% N) was applied in three doses as per recommendation of the university (Table 1).

### 2.2. Description and Mounting of N-Sensor

**Figure 2:**
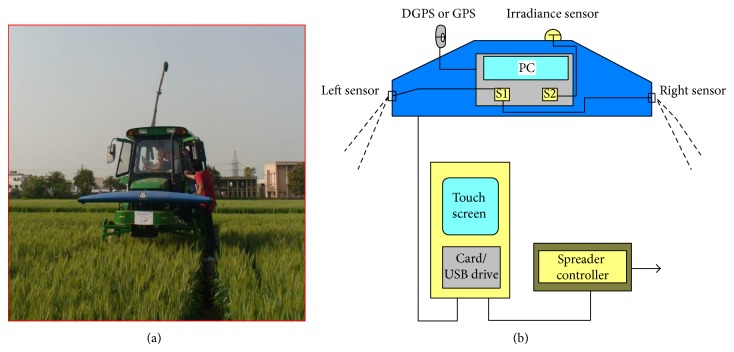
N-sensor (a) in wheat crop installed over the multiutility high clearance vehicle, (b) different parts with setup.

The N-sensor was mounted on the front of a multiutility vehicle (MUV) developed in the department [18] at 1.6 m above the ground, Figure 2. Data collection at all growth stages can be performed by using MUV in crops due to having almost double ground clearance compared to the normal tractor. It has also narrow rear tyre width (20 cm), which causes lesser crop damage. It has air conditioner cabin, providing the adequate working temperature to the operator or sophisticated instruments. Different sensors, like N-sensor, multispectral camera, and so forth, can be mounted easily on this for collection of real time data in standing field crops requiring a plant-level spatial resolution. MUV can easily be lowered down to operate as a normal tractor.

**Figure 3:**
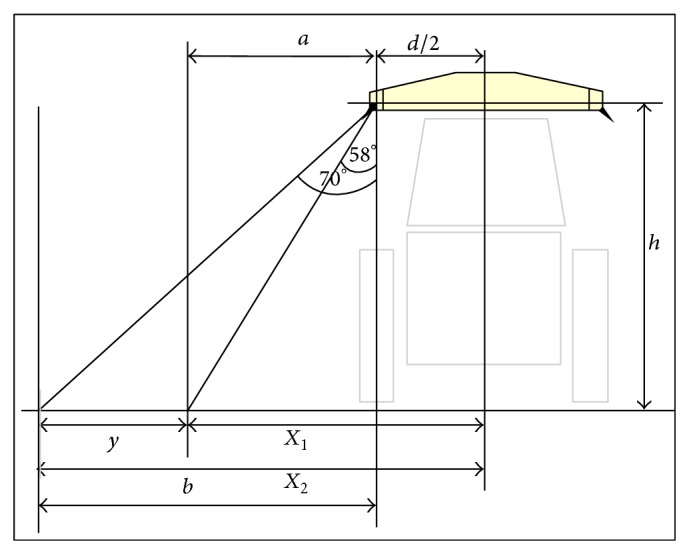
Front geometry of N-sensor mounted over the vehicle.

N-sensor consists of two diodes array spectrometers, Figure 3. One spectrometer analyses crop light reflectance received by four lenses with an oblique view on to the crop (two on each side of the vehicle). The second spectrometer is used to measure the irradiance of ambient light for permanent correction of the reflectance signal to ensure stable measurements with changing irradiance conditions. Two strips can be scanned on both sides of the vehicle during its movement in the crop. The N-sensor measures light reflectance from the crop from four different angles. Measurements are taken continuously with the system designed to operate at normal working speeds. The N-sensor system is connected to a GPS signal to allow location of sensor and application information to be plotted enabling the production of “biomass” and “nitrogen” application maps for the field.

One pass of the field scan was done for each plot in the wheat fields. For each measurement, the field scan recorded spectral information at all wavebands from 600 to 1100 nm with 10 nm intervals. N-sensor scans were done at Tillering (Growth stage: 55DAS or 28 for N-sensor), Booting (growth stage: 85DAS or 36 for N-sensor) and ear emergence (growth stage: 115DAS or 50 for N-sensor) stages of crop growth.

The nitrogen and biomass status of crop were also mapped during scanning of crops at different growing period based on spectral reflectance measurement.

### 2.3. Geometry of N-Sensor

The area scanned by the N-sensor depends on the height of mounting over the tractor. In present case, N-sensor was mounted at 1.6 m height from the ground. Equations (1) [19] can be obtained through which area sensed by the N-sensor can be calculated as follows (Figure 3):

(1) $$\begin{array}{c} X_{1}=\frac{d}{2}+\frac{h\tan58^{{}^{\circ}}}{{\left(2\right)}^{1/2}}=0.5d+1.13h,\\ X_{2}=\frac{d}{2}+\frac{h\tan70^{{}^{\circ}}}{{\left(2\right)}^{1/2}}=0.5d+1.94h, \end{array}$$

where *d* is length of the sensor rig (2.0 m), *h* is height of sensor rig, *X*_1_ is inner point of sensed area, *X*_2_ is outer point of sensed area, *y* is width of sensed area, m (*X*_2_ − *X*_1_), and *h* is height of sensor mounting, 1.6 m.

From (1), *X*_1_ = 2.831 m and *X*_2_ = 4.143 m, *y* = *X*_2_ − *X*_1_ = 1.32 m, total width on both sides of tractor = 8.28 m,  and total width of scan by spectrometers = 2.64 m.

When sensor was mounted at height (*h*) of 1.6 m, it is scanning width of (*y*) 1.32 m or 2.64 m on both sides of a tractor covering total width 8.28 m. It means spectrometers can scan about 32% of total area of crop under consideration.

### 2.4. N-Sensor Calibration

Tractor mounted N-sensor was calibrated at three important crop growth stages, that is, tillering, booting, and ear emergence. Tractor mounted with N-sensor was operated in plots having different N treatments, that is, 0, 30, 60, 90, 120, and 150 kg N/ha. Different sensor attributes like sensor SN values, biomass data, and most common Normalized Difference Vegetation Index (NDVI) were determined from the N-sensor spectral data collected in the range of 400–1050 nm measured during the field calibration.

### 2.5. Agronomic Data Collection

The data for various parameters like total chlorophyll and nitrogen content were measured in laboratory at different growth stages like tillering, booting, and ear emergence of crop and at the same time N-sensor data was also recorded. The chlorophyll content was estimated according to the method of Hiscox and Israelstam [20] using dimethyl sulphoxide (DMSO) chemical in laboratory conditions. Nitrogen present in the plant leaves was calculated by using titration method in laboratory.

### 2.6. Development of Sensor Algorithm

By establishing a reference strip, sensor readings can be collected under near perfect conditions (in terms of crop health). These readings are considered the optimum sensor reflectance value, a value at which no additional N is required. From these sensor readings a “Sufficiency Index” (SI) can be calculated using the following equation:

(2) $$\begin{array}{c} \text{SI}=\frac{\text{Target}\;\text{Reflectance}}{\text{Reference}\;\text{Reflectance}}. \end{array}$$

The SI value is a measure of N sufficiency. An SI value of 1 means the crop is N sufficient and no N is needed, and an SI value of less than 1 means the crop is deficient and additional N is required (more N needed as the SI value decreases). For example, if the reference strip reflectance value is more target area reflectance value is low. When SI value is calculated it will be small, meaning the target area is N deficient and more N will be required.

The procedures for development of sensor algorithm for translating sensor readings into N applications (3) are outlined as follows:

(3) $$\begin{array}{c} {\text{SI}}_{\text{sensor NDVI}}=\frac{\text{NDVI}}{{\text{NDVI}}_{\text{N reference}}}. \end{array}$$

Normalizing sensor data to a well fertilized reference plot allows the estimation of the crop's ability to respond to applied N and serves to normalize data to a particular environment. This algorithm was to be developed on the SPAD based framework [17]; the generalized and specific equations form of the quadratic (second order polynomial) response functions are

(4) $$\begin{array}{c} {\text{SI}}_{\text{SPAD}}=a_{0}+a_{1}*{\text{N}}_{\text{rate}}+a_{2}*{\left({\text{N}}_{\text{rate}}\right)}^{2}. \end{array}$$

For (4), the *a*_0_, *a*_1_, *a*_2_ coefficients represent the intercept, linear, and quadratic terms, respectively, and the response portion of the equation can be solved for N rate as follows:

(5) $$\begin{array}{c} {\text{N}}_{\text{rate}}\left({\text{SI}}_{\text{SPAD}}\right)=\frac{-a_{1}-\left(\sqrt{{a_{1}}^{2}-4a_{2}\left(a_{0}-{\text{SI}}_{\text{SPAD}}\right)}\right)}{2a_{2}}. \end{array}$$

In this case, yield maximizing N_rate_ can be found as

(6) $$\begin{array}{c} {\text{N}}_{\text{rate}}\left(\max{\text{SI}}_{\text{SPAD}}\right)=\frac{{-a}_{1}}{{2a}_{2}}. \end{array}$$

**Figure 4:**
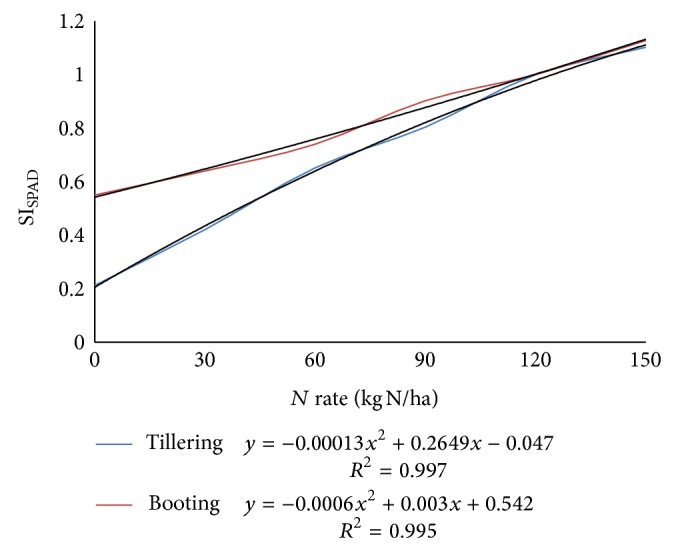
Quadratic response model from regression analyses of relative chlorophyll meter readings (SI_SPAD_) and N fertilizer rate at tillering and booting stages.

For (6), the N_rate_ that corresponded to the maximum SI_SPAD_ measurement is 150 kg N ha^−1^ (Figure 4). An appropriate nitrogen application rate (N_app_) for any SI_SPAD_ measurement below maximum (max SI_SPAD_) can then be found as follows:

(7) $$\begin{array}{c} {\text{N}}_{\text{app}}={\text{N}}_{\text{rate}}\left(\max{\text{SI}}_{\text{SPAD}}\right)-{\text{N}}_{\text{rate}}\left({\text{SI}}_{\text{SPAD}}\right). \end{array}$$

Next, the method for converting sufficiency indices that are based on the canopy sensor measurements (SI_sensor  NDVI_) into SI_SPAD_ measurements for input into the SPAD algorithm is illustrated. Solari et al. [21] demonstrated a linear association between SI_SPAD_ and SI_sensor_ illustrating its general form as

(8) $$\begin{array}{c} {\text{SI}}_{\text{SPAD}}=b_{0}+b_{1}*{\text{SI}}_{\text{sensor}}. \end{array}$$

For (8), *b*_0_, *b*_1_ coefficients represent the *y* intercept and linear terms, respectively. Using the same data set, the following specific equation was determined for SI_NDVI_. Equation (8) was used in development of the sensor algorithm, which hereafter is referrred to as SI_sensor_. After substituting (5), (6), and (8) into (7), the following generalized parametric function can be derived:

(9) $$\begin{array}{c} {\text{N}}_{\text{app}}=R\sqrt{{\text{SI}}_{R}-{\text{SI}}_{\text{sensor}}}, \end{array}$$

where *R* and SI_*R*_ terms can be solved for using the following equations:

(10) $$\begin{array}{c} R=\sqrt{\frac{{-b}_{1}}{a_{2}}},\\ {\text{SI}}_{R}=\frac{4a_{2}a_{0}-{a_{1}}^{2}-4a_{2}b_{0}}{4a_{2}b_{1}}. \end{array}$$

**Figure 5:**
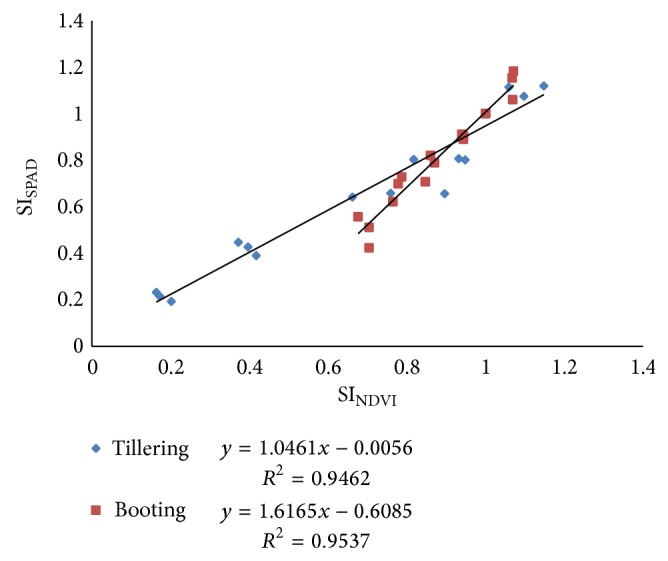
Relationship between relative variation in sensor determined vegetation index (SI_NDVI_) and variation in relative chlorophyll meter (SI_SPAD_) readings for data collected during tillering and booting stages.

Quadratic response model from regression analyses of relative chlorophyll meter readings (SI_SPAD_) and N fertilizer rate at tillering and booting stages of wheat crop are shown in Figure 4. Relationships between relative variations in sensor determined vegetation indices (SI_NDVI_) and variation in relative chlorophyll meter (SI_SPAD_) readings for data collected at tillering and booting stages are given in Figure 5.

*For Tillering Stage*. From Figures 4 and 5,

(11)  SISPAD=−0.047+0.2649Nrate−0.00013Nrate2, SISPAD=−0.0056+1.0461∗SIsensor NDVI.

Substitution of coefficients from (11) into (9) results in the sensor algorithm taking the following specific form:

(12) $$\begin{array}{c} {\text{N}}_{\text{app}}=87.17\sqrt{1.35-{\text{SI}}_{\text{sensor}}}. \end{array}$$

*For Booting Stage*. Again from Figures 4 and 5,

(13)  SISPAD=0.542+0.003Nrate−0.0006Nrate2, SISPAD=−0.608+1.616∗SIsensor  NDVI.

Substitution of coefficients from (13) into (9) results in the sensor algorithm taking the following specific form:

(14) $$\begin{array}{c} {\text{N}}_{\text{app}}=51.89\sqrt{1.33-{\text{SI}}_{\text{sensor}}}. \end{array}$$

### 2.7. N-Recommendation

N-recommendation at tillering and booting stages of crop growth was given in two ways, first on the basis of sensor value (SN) values measured during the calibration of N-sensor and second on the basis of algorithm developed in Section 2.6. In algorithm developed, N_app_ (N-application) rate can be determined through input of SI_sensor_ values into the simplified equations (12) and (14) for tillering and booting stages, respectively, which represents the algorithm for translating sensor reading into N-application rates based on crop N sufficiency.

### 2.8. Statistical Analysis

Various data sets were statistically analyzed by general linear model (GLM) procedure by using SAS software 9.3. All possible pairs of treatment means were compared at 5% level of significance. Pearson's correlation coefficients (*r*) among variables were determined by the CORR procedure in the SAS system (SAS Institute, Cary, NC, USA).

## 3. Results and Discussion

Tractor mounted N-sensor was calibrated for the determination of N and chlorophyll content available in wheat crop at different growth stages, that is, tillering, booting, and ear emergence. The relationships among different sensor attributes like sensor values (SN), biomass data of the crop, and NDVI calculated from the sensor spectral data were established with N and chlorophyll content in the leaves of crop. Results are discussed regarding the N-recommendation for wheat crop on the basis of sensor values and algorithm developed in the study.

### 3.1. Relationship among N-Sensor Attributes with Different N-Application Rate

**Figure 6:**
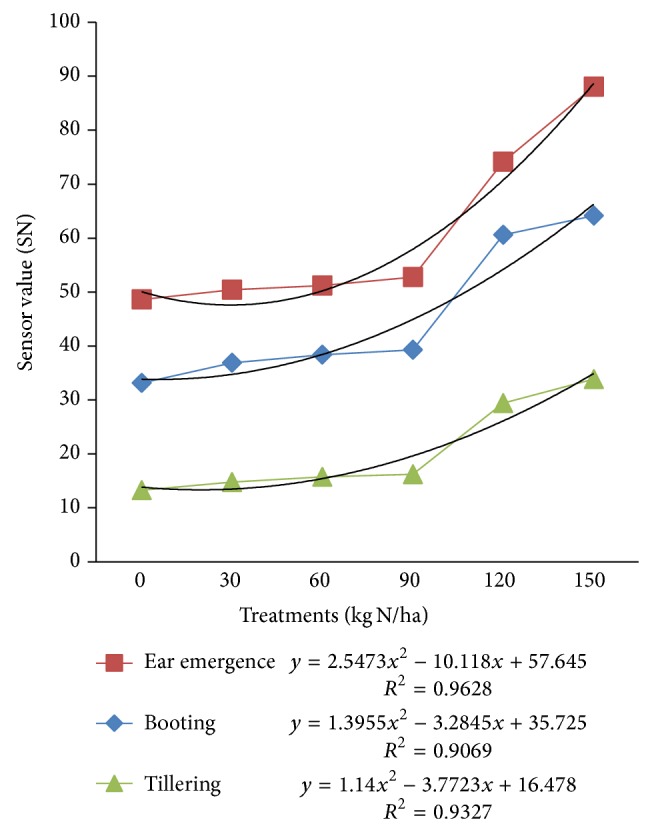
Sensor values for different N-treatments at different crop growth stages.

Figure 6 shows the sensor values (SN) obtained with different nitrogen treatments in wheat crop at its different growth stages. Increasing sensor value from date to date at a given N rate and more distinct differences between N rates at the different growth stages reflect the growth and N uptake pattern of the crop. There was a strong polynomial relationship between sensor value (SN) and N-treatments with coefficients of determination ranging from 0.90 to 0.96 (*P* < 0.001).

**Figure 7:**
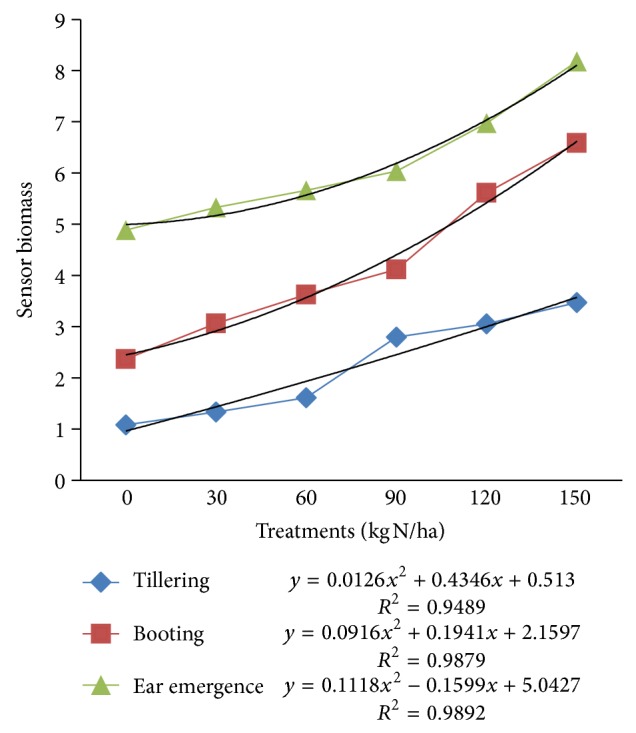
Biomass values for different N-treatments at different crop growth stages.

The relationships between sensor biomass values across N application rates at different growth stages are shown in Figure 7. There was also a strong polynomial relationship between sensor biomass values and N-treatments with coefficients of determination ranging from 0.94 to 0.98 (*P* < 0.001).

**Figure 8:**
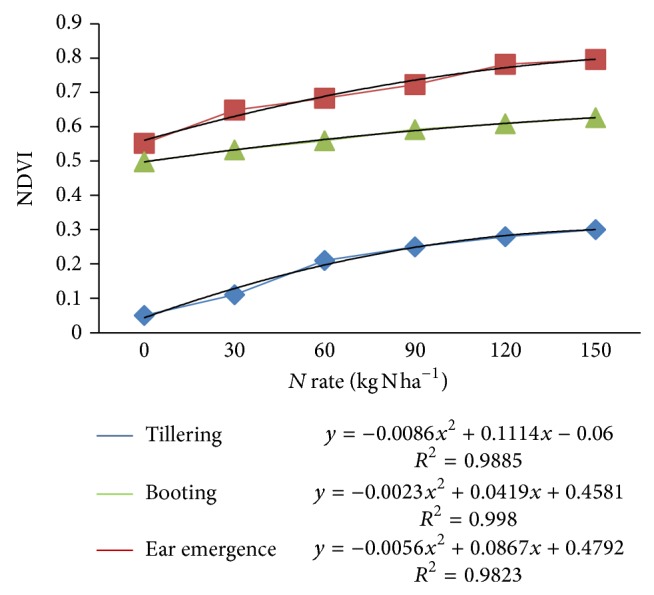
Relation between NDVI values calculated from N-sensor data for different N-treatments at different crop growth stages.

The NDVI calculated from sensor spectral data across N application rates at different growth stages are shown in Figure 8. However, it was determined that coefficients of determination of NDVI with applied N rate would be the best indicator of sensor performance, and thus comparisons were based on this analysis. At all wheat growth stages, sensor showed increasing NDVI values for different N-levels. There was also strong polynomial relationship between NDVI and N rates with coefficients of determination ranging from 0.98 to 0.99 (*P* < 0.001). This is in accordance with many studies conducted to recommend N on the basis of NDVI calculated from crop reflectance data.

### 3.2. Relationship among N-Sensor Attributes and Different Crop Growth Parameters

The correlation coefficients corresponding to the relationships between NDVI calculated from N-sensor data, biomass value given by sensor, sensor value (SN) with plant N concentration, chlorophyll content of leaves at three growth stages, and yield were obtained from experiments. These relationships have been discussed in the following subheads.

#### 3.2.1. Relationship among N-Sensor Attributes and Lab *N*

**Figure 9:**
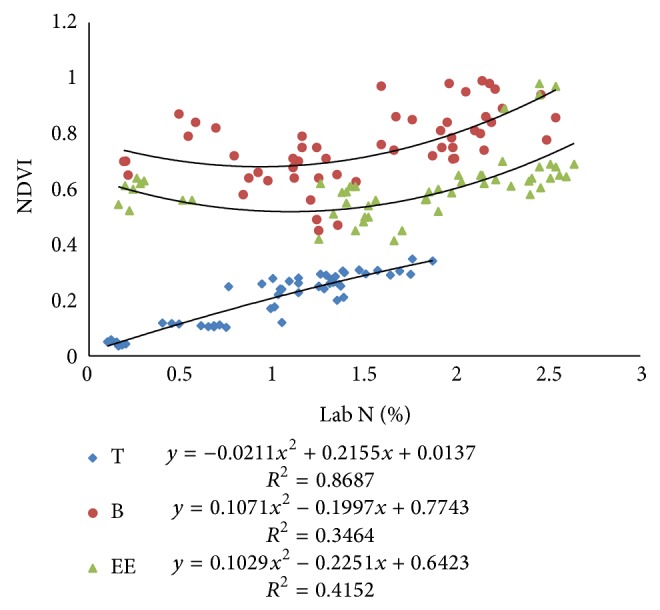
Correlations between NDVI calculated from N-sensor data and Lab N at different crop growth stages.

**Figure 10:**
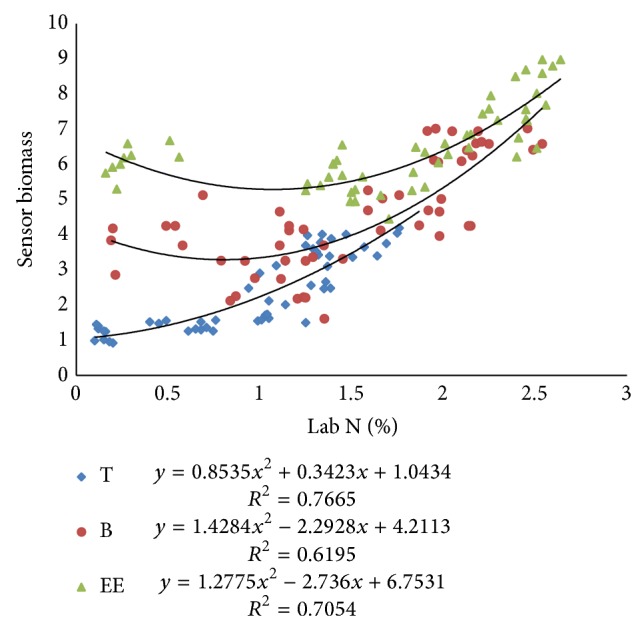
Correlations between sensor biomass and Lab N at different crop growth stages.

**Figure 11:**
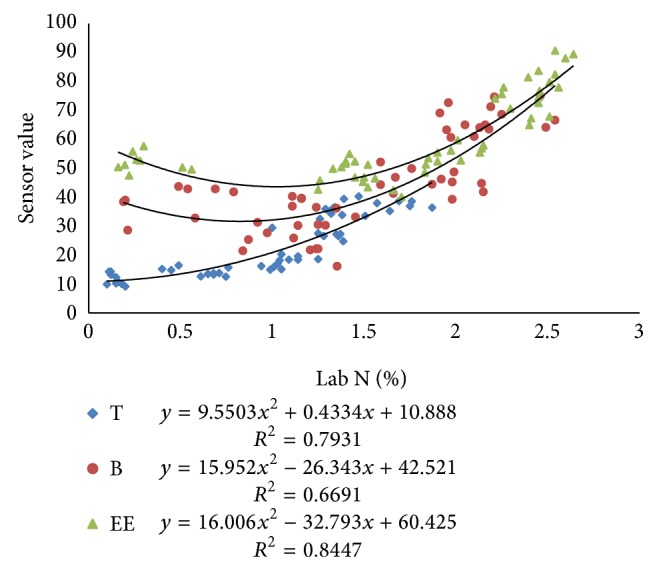
Correlations between sensor values (SN) and Lab N at different crop growth stages.

**Figure 12:**
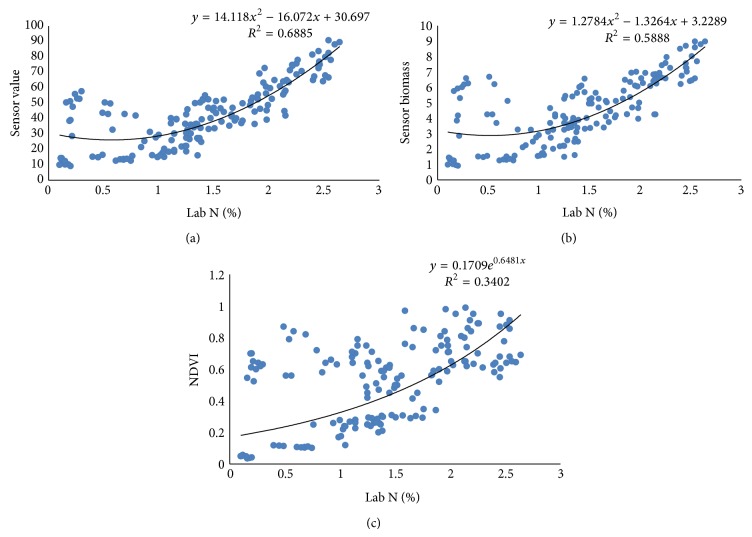
Correlations between different N-sensor attributes and Lab N for complete crop growth.

Pearson correlation coefficients (PCC) between Lab N and N-sensor attributes were more at tillering stage as compared to the values at booting and ear emergence stages of crop growth (Table 2). The Pearson correlation coefficients between sensor biomass with Lab N were higher for tillering stage, that is, 0.87. Similarly, the correlation coefficient between NDVI of sensor and Lab N was higher for tillering stage, that is, 0.90. The correlation coefficient between sensor value and Lab N was higher for first stage, that is, 0.85. There were also good polynomial relationships between sensor attributes and Lab N (Figures 9, 10, and 11) especially at tillering stage. Sensor attributes are well correlated with Lab N at tillering stage as compared to the booting and ear emergence stages of the crop growth. Correlation among N-sensor attributes and Lab N for complete crop growth is also shown in Figure 12. Overall sensor value is a good indicator for the Lab N prediction at all growth stages of the crop.

#### 3.2.2. Relationship among N-Sensor Attributes and Lab Chl

**Figure 13:**
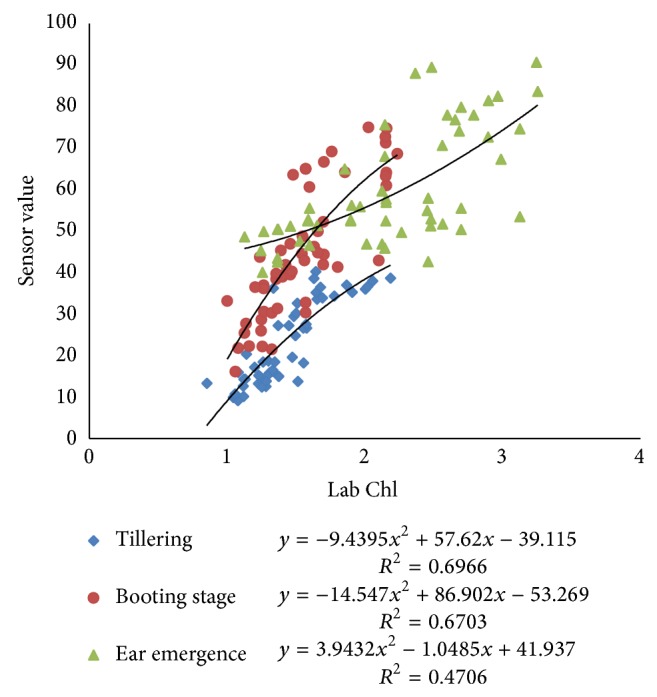
Correlations between sensor values (SN) and Lab Chl at different crop growth stages.

**Figure 14:**
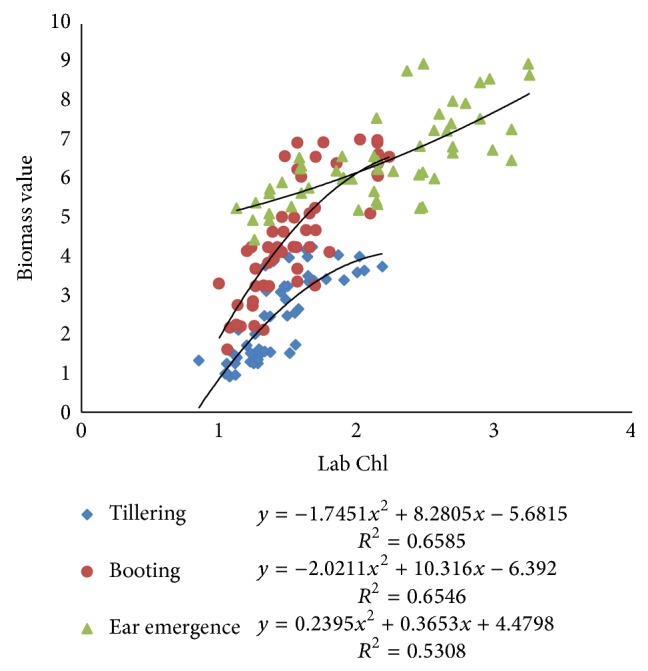
Correlations between sensor biomass values and Lab Chl at different crop growth stages.

**Figure 15:**
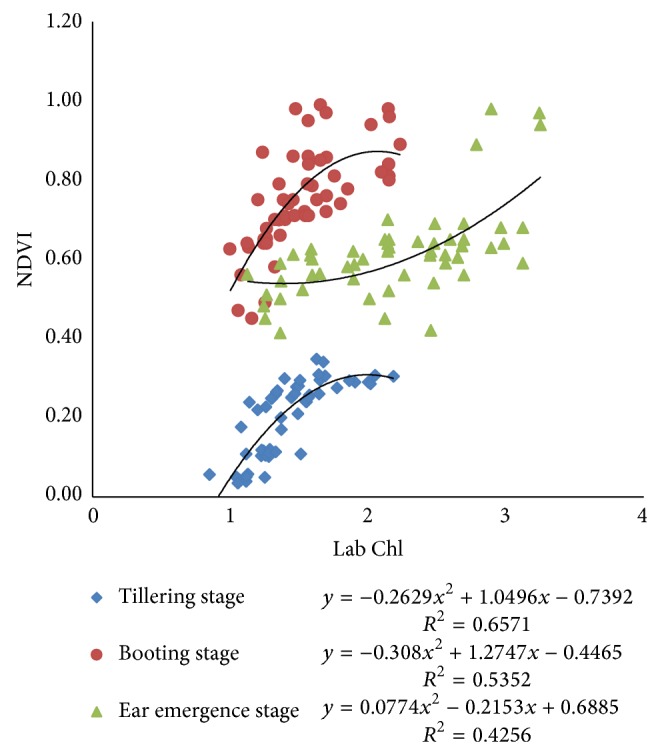
Correlations between NDVI calculated from N-sensor data and Lab Chl at different crop growth stages.

**Figure 16:**
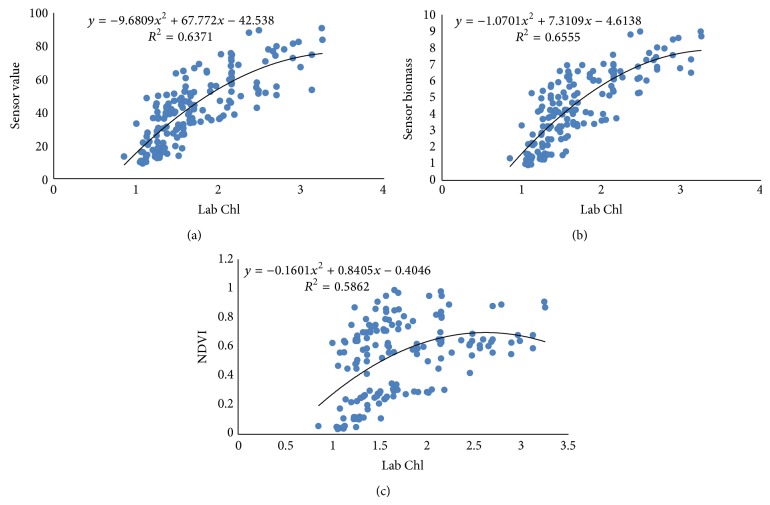
Correlations between different sensor attributes and Lab Chl for complete crop growth.

Pearson correlation coefficients between N-sensor attributes and Lab Chl were ranging from 0.61 to 0.82 at all growth stages of crop. The Pearson correlation coefficient between sensor value and Lab Chl was higher for both tillering and booting stage, that is, 0.82 and 0.81. The correlation between sensor biomass and Lab Chl decreased with the growth stages. The coefficient values were 0.79, 0.79, and 0.72 for tillering, booting, and ear emergence stages, respectively. The sensor NDVI value correlation with Lab Chl was having values of PCC 0.75, 0.67, and 0.61 at tillering, booting, and ear emergence stages, respectively (Table 3). Polynomial relationships between sensor attributes and Lab Chl are shown in Figures 13, 14, and 15. Figures indicate that the relationships among different sensor attributes are poorly correlated with Lab Chl data as compared with Lab N data. Correlation among N-sensor attributes and Lab Chl or complete crop growth is also shown in Figure 16. Overall sensor value is a good indicator for prediction of Lab Chl at all growth stages of the crop.

#### 3.2.3. Relationship among N-Sensor Attributes and Crop Yield

Correlation between yield and sensor attributes at different growth stages of crop is shown in Table 4. Table indicates that there is a good correlation between sensor attributes and yield at all growth stages of the crop. But tillering stage is most prominent stage to predict crop yield as compared to the other stages by using sensor attributes.

### 3.3. Nitrogen Application/Recommendation

#### 3.3.1. Based upon Sensor Algorithm

**Figure 17:**
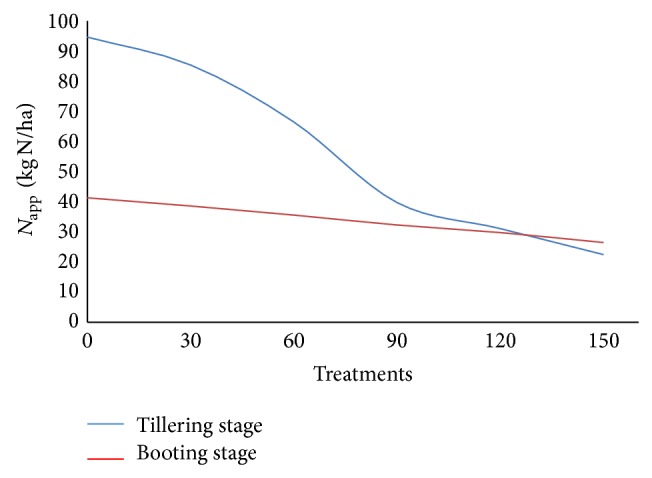
N-application (N_app_ rate) as a function of different N-treatments using N-sensor algorithm during tillering and booting stages.

N-application (N_app_ rate) as a function of different N-treatments using N-sensor algorithm developed for tillering and booting stages is shown in Figure 17. It is clearly indicated that algorithm developed for tillering and booting stages is useful for the prediction of N-application rates for wheat crop. Figure shows that during tillering stage 95 kg N/ha should be applied to the plot having 0 kg N/ha and 42 kg N/ha should be applied to the plot with 150 kg N/ha. Similarly at booting stage 42 kg N/ha is to be applied in plot with 0 kg N/ha and 25 kg N/ha should be applied in plot already having 150 kg N/ha.

#### 3.3.2. Based upon Sensor Value

**Figure 18:**
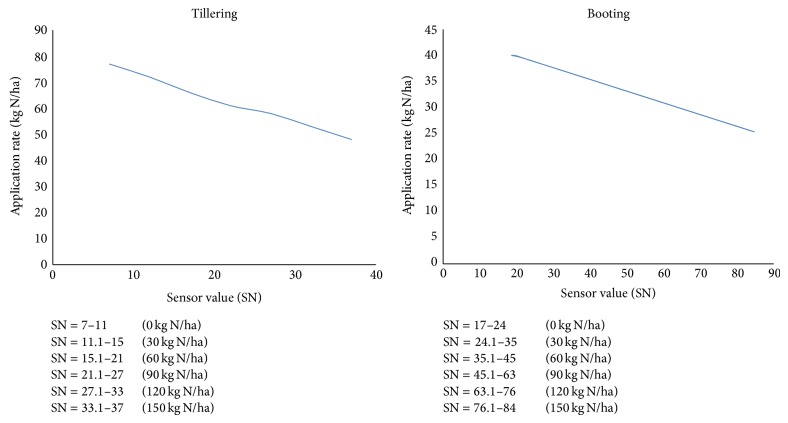
N-application (N_app_) rate on the basis of N-sensor values (SN) during tillering and booting stages of wheat crop.

N-application (N_app_) rates on the basis of N-sensor values (SN) during tillering and booting stages of wheat crop are shown in Figure 18. Figure shows that with the increase in sensor values N-applied rate decreases. During tillering stage, 75 kg N/ha should be applied in the plot having 0 kg N/ha corresponding to the sensor values ranging from 7 to 11 as compared to 45 kg N/ha which should be applied in the plot with 150 kg N/ha corresponding to the sensor values ranging from 33.1 to 37. Similarly at booting stage, 40 kg N/ha is to be applied in plot with 0 kg N/ha corresponding to SN values ranging from 17 to 24 as compared to 25 kg N/ha which should be applied in plot already having 150 kg N/ha with SN values 76.1–84.

It is clear that N-application rates based upon algorithm and sensor value methods were in consonance. Both of the methods predicted almost the same application rates for plots with different levels of N applied.

## 4. Conclusions

- For tractor mounted N-sensor, spectrometers can scan about 32% of total area of crop under consideration.
- Algorithms were developed for N-application at tillering and booting stages of crop growth.
- There was a strong correlation among sensor attributes and different N-levels, that is, 0, 30, 60, 90, 120, and 150 kg N/ha.
- Study indicates that the relationship among different sensor attributes is poorly correlated with Lab Chl data as compared with Lab N data.
- It was concluded that there is a good correlation between sensor attributes and yield at all growth stages of the crop. But tillering stage is most prominent stage to predict crop yield as compared to the other stages by using sensor attributes.
- The algorithms developed for tillering and booting stages are useful for the prediction of N application rates for wheat crop.
- N-application rates predicted by algorithm developed and sensor value were almost the same for plots with different levels of N applied.

## Figures and Tables

**Table 1:** Fertilizer in form of urea application timings and doses.

Treatments	Dose (KgN/ha)			Total (kgN/ha)
	Basal (50%)	At 1st irrigation (25%)	At 2nd irrigation (25%)	
N1	0	0	0	0
N2	15	7.5	7.5	30
N3	30	15	15	60
N4	45	22.5	22.5	90
N5	60	30	30	120
N6	75	37.5	37.5	150

**Table 2:** Pearson correlation coefficients among different sensor attributes and Lab N at different growing stages of crop.

Relationship between	Tillering	Booting	Ear emergence	Overall
Sensor value and Lab N	0.85	0.63	0.64	0.68
	<0.001	<0.001	<0.001	

Sensor biomass and Lab N	0.87	0.610	0.57	0.58
	<0.001	0.0067	0.0132	

Sensor NDVI and Lab N	0.90	0.49	0.41	0.34
	<0.001	0.0364	0.0872	

**Table 3:** Pearson correlation coefficients among different sensor attributes and Lab Chl at different growing stages of crop.

Relationship between	Tillering	Booting	Ear emergence	Overall
Sensor value and Lab Chl	0.82	0.81	0.67	0.63
	<0.001	<0.001	<0.001	

Sensor biomass and Lab Chl	0.79	0.79	0.72	0.65
	<0.001	<0.001	<0.001	

Sensor NDVI and Lab Chl	0.75	0.67	0.61	0.58
	<0.001	<0.001	<0.001	

**Table 4:** Pearson correlation coefficients among sensor attributes at different growth stages and crop yield.

Relationship between	Tillering	Booting	Ear emergence
Sensor value and yield	0.94	0.85	0.85
	<0.001	<0.001	<0.001

Sensor biomass and yield	0.94	0.85	0.85
	<0.001	<0.001	<0.001

Sensor NDVI and yield	0.92	0.87	0.72
	<0.001	<0.001	0.0006

## Abbreviations

CCI: Chlorophyll Concentration Index
Lab N: Nitrogen in leaves of wheat determined in laboratory
CC: Chlorophyll content in leaves of wheat determined in laboratory
PC: Pearson's coefficient
RCBD: Randomized completely block design
N: Nitrogen
T: Tillering stage
B: Booting stage
EE: Ear emergence stage.
